# Specific immunotherapy using *Hymenoptera* venom: systematic review

**DOI:** 10.1590/S1516-31802010000100007

**Published:** 2010-01-07

**Authors:** Alexandra Sayuri Watanabe, Luiz Augusto Marcondes Fonseca, Clóvis Eduardo Santos Galvão, Jorge Kalil, Fabio Fernandes Morato Castro

**Affiliations:** I MD, MSc. Allergist and immunologist in the Discipline of Allergy and Clinical Immunology, Universidade de São Paulo (USP), São Paulo, Brazil.; II MD. Attending physician in the Clinical Immunology and Allergy Service, Hospital das Clínicas (HC), São Paulo, Brazil.; III MD. Director and professor of Clinical Immunology and Allergy Service, Hospital das Clínicas (HC), São Paulo, Brazil.; IV MD. Supervisor and associate professor of Clinical Immunology and Allergy Service, Hospital das Clínicas (HC), São Paulo, Brazil.

**Keywords:** Hymenoptera, Insects, Immunotherapy, Meta-analysis [publication type], Review [publication type], Himenópteros, Insetos, Imunoterapia, Metanálise, Revisão

## Abstract

**CONTEXT AND OBJECTIVE:**

The only effective treatment for patients who have severe reactions after *Hymenoptera* stings is venom immunotherapy. The aim of this study was to review the literature to assess the effects of venom immunotherapy among patients presenting severe reactions after *Hymenoptera* stings.

**DESIGN AND SETTING:**

Randomized controlled trials in the worldwide literature were reviewed. The manuscript was produced in the Discipline of Allergy and Clinical Immunology, Universidade de São Paulo (USP).

**METHODS:**

Randomized controlled trials involving venom immunotherapy versus placebo or only patient follow-up were evaluated. The risk of systemic reactions after specific immunotherapy was evaluated by calculating odds ratios (OR) and their 95% confidence intervals.

**RESULTS:**

2,273 abstracts were identified by the keywords search. Only four studies were included in this review. The chi-square test for heterogeneity showed that two studies were homogeneous and could be included in a meta-analysis. By combining the two studies, the odds ratio became significant: 0.29 (0.10-0.87). However, analysis on the severity of the reactions after immunotherapy showed that the benefits may not be so significant because the reactions were mostly similar to or milder than the original reaction.

**CONCLUSIONS:**

Specific immunotherapy should be recommended for adults and children with moderate to severe reactions, but there is no need to prescribe it for children with skin reactions alone, especially if the exposure is very sporadic. On the other hand, the risk-benefit relation should always be assessed in each case.

## RESUMO

**CONTEXTO E OBJETIVO:**

O único tratamento eficaz para pacientes que têm reações graves após ferroada de *Hymenoptera* é a imunoterapia com veneno. O objetivo deste estudo foi rever a literatura para avaliar os efeitos da imunoterapia com veneno em pacientes com reações graves após ferroada de *Hymenoptera*.

**TIPO DE ESTUDO E LOCAL:**

Foram revisados na literatura mundial ensaios clínicos controlados e aleatórios. O manuscrito foi realizado na Disciplina de Alergia e Imunologia Clínica, Universidade de São Paulo (USP).

**Métodos:**

Ensaios clínicos controlados e aleatórios envolvendo imunoterapia com veneno de *Hymenoptera versus* placebo ou apenas acompanhamento dos pacientes foram avaliados. Realizada imunoterapia específica, o risco de reações sistêmicas foi avaliado através de cálculo do “*odds ratio*” e intervalo de confiança de 95%.

**RESULTADOS:**

2.273 resumos foram identificados na busca pelas palavras chave. Apenas quatro estudos foram incluídos nesta revisão. O teste qui-quadrado de heterogeneidade mostrou que dois estudos foram homogêneos e puderam ser incluídos na metanálise. Ao combinar os dois estudos, o “*odds ratio*” passou a ser significativo: 0.29 (0.10-0.87). Entretanto, ao analisar a gravidade das reações ocorridas após a imunoterapia, observou-se que os benefícios podem não ser tão relevantes, pois as reações foram, na grande maioria, ou mais leves ou semelhantes à reação original.

**CONCLUSÕES:**

A imunoterapia específica deve ser recomendada para adultos e crianças com reações moderadas a graves, porém não há necessidade de prescrevê-la para as crianças apenas com reações cutâneas, especialmente se a exposição é muito esporádica. No outro lado, a relação risco-benefício deve ser sempre avaliada em cada caso.

## INTRODUCTION

Insects of the order *Hymenoptera* (bees, wasps and ants) have been exhaustively studied in medicine, not only because of the therapeutic possibilities of venom components, but also because of the intensity and severity of the allergic reactions that their venom causes. Drugs, foods, latex and *Hymenoptera* venom are the most commonly reported culprits for anaphylaxis.^1^ Recent studies have suggested that the prevalence of *Hymenoptera* sting allergy is about 0.4% to 3.3%.^2^

The clinical manifestations produced by *Hymenoptera* venom can be didactically classified into allergic reactions (extensive local reaction, anaphylactic systemic reaction and late systemic reaction) and non-allergic reactions (local reaction and toxic systemic reaction).^3,4^

The treatment for systemic allergic reactions to *Hymenoptera* venom consists of emergency treatment and specific allergen immunotherapy, which is considered to be a safe and effective treatment.^5^ However, its real success rate seems to vary between different centers, because the results are based on non-controlled studies and are normally limited by the sample size and the low statistical power.

Therefore, a systematic review of the worldwide literature is very important from the scientific point of view, in order to critically assess all the studies already produced, and to organize and compare data using systematic methods.

## OBJECTIVES

### The objectives of the present study were:

To perform a systematic review of published and non-published studies in the worldwide literature on *Hymenoptera* venom immunotherapy among patients who presented systemic allergic reactions after insect stings. Therefore, all randomized trials comparing *Hymenoptera* venom immunotherapy with placebo or emergency treatment (for example, epinephrine autoinjection) would be identified;

To assess the methodological quality of these randomized controlled trials;

To determine the heterogeneity of the studies, and analyze possible differences among the several types of treatment.

## METHODS

### Types of studies

Any randomized controlled trials examining treatment given to patients who presented systemic allergic reactions to *Hymenoptera* stings, in which immunotherapy was administered by injection, were eligible for inclusion in this review. Other administration routes, such as sublingual or oral, were excluded. Only randomized controlled trials were included, in order to reduce the bias that is associated with other types of studies. The allergen extracts administered were: honeybee, wasp (yellow jacket, white hornet, yellow hornet and *Polistes* sp.) and ant (*Solenopsis invicta* and *Myrmecia pilosula*). The co-interventions included placebo or emergency treatment (follow-up alone for patients who were given guidance for adrenaline self-administration, using an epinephrine autoinjector). The trials were included even if they were single-blind.

### Types of participants enrolled in the studies

The participants were children and adults of both sexes with *Hymenoptera* venom hypersensitivity, as defined by a history of systemic anaphylactic reaction after insect stings and the presence of a positive skin test (prick or intradermal test).

### Types of intervention

*Hymenoptera* venom immunotherapy was compared with placebo and with no treatment (follow-up or use of an epinephrine self-administration device. i.e. an epinephrine autoinjector).

The following items were accepted as exclusion criteria: history of systemic hypertension, heart disease or poorly controlled lung disease; and use of angiotensin-converting enzyme (ACE) inhibitors, beta-blocker therapy or a negative skin test.

### Types of outcome measurements

– Change in clinical manifestation after sting challenge or accidental stings.
– Indication for venom immunotherapy.
– Change in levels of venom-specific immunoglobulin E (IgE) or immunoglobulin G (IgG) antibodies.

### Search strategy to identify studies

- *Strategy to locate randomized controlled trials*
- *Strategy to locate the insect terms*
  bees.mp. or exp BEES/
  bee venom.mp. or exp Bee Venoms/
  honeybee.mp.
  hymenoptera.mp. or exp HYMENOPTERA/
  wasps.mp. or exp WASPS/
  wasp venom.mp. or exp Wasp Venoms/
  white hornet.mp.
  yellow jacket.mp.
  yellow hornet.mp.
  polistes.mp.
  ants.mp. or exp ANTS/
  ant venom.mp. or exp Ant Venoms/
  solenopsis invicta.mp.
  myrmecia pilosula.mp.
- *Strategy to locate types of reactions*
  exp “Insect Bites and Stings”/
  exp HYPERSENSITIVITY, DELAYED/ or exp HYPERSENSITIVITY/ or hypersensitivity.mp. or exp HYPERSENSITIVITY, IMMEDIATE/
  exp Anaphylaxis/ or anaphylactic.mp.
  (allergic or allergy).mp.
  swelling.mp.
  exp EDEMA/ or edema.mp.
  systemic reaction.mp.
  shock.mp. or exp SHOCK, TRAUMATIC/ or exp SHOCK/
  exp INFLAMMATION MEDIATORS/ or inflammation.mp. or exp INFLAMMATION/
  hives.mp. or exp Urticaria/
  exp Airway Obstruction/ or angiodema.mp.
  laryngeal obstruction.mp.
  bronchospasms.mp. or exp Bronchial Spasm/
  gastro-intestinal symptoms.mp.
  vasculitis.mp. or exp VASCULITIS, ALLERGIC CUTANEOUS/ or exp VASCULITIS/ or exp VASCULITIS, HYPERSENSITIVITY/
  glomerulonephritis.mp. or exp GLOMERULONEPHRITIS/
  polyradiculitis.mp. or exp Polyradiculopathy/
  exp DEATH, SUDDEN/ or exp DEATH/ or death.mp.
- *Strategy to locate types of reactions*
  exp IMMUNOTHERAPY, ACTIVE/ or immunotherapy.mp. or exp IMMUNOTHERAPY/
  adrenaline.mp. or exp Epinephrine/
  exp Injections/ or epipen.mp.

Searches were conducted to identify randomized controlled trials, in any language, going from the dates when the following databases began: Medline (Medical Literature Analysis and Retrieval System Online), Lilacs (Literatura Latino-Americana e do Caribe em Ciências da Saúde), Embase (Excerpta Médica Database), SciSearch (Science Citation Index Expanded), SciELO (Scientific Electronic Library Online) databases and the Cochrane Database of Systemic Reviews. Reference lists of recent reviews and published trials were also searched.

The decisions on whether to include studies in the review were made through discussion between two of the review authors (ASW and LAMF), after all of the studies had been read by ASW. For trials that seemed to meet the inclusion criteria or for which there was insufficient information in the title and abstract to make a clear decision, the full reports were obtained.

Each of the suitable reports was read in detail by ASW, and relevant information was abstracted onto a standard extraction sheet (covering study type and methodology, number and description of subjects, details of type, dosage and time schedule/duration of intervention, type, timing and measurement method for outcomes). All data were extracted and ASW and LAMF independently assessed the methodological quality of the studies included, using a validated quality checklist.^6^ This instrument could only assess some items and its use to include or exclude systematic review studies is not recommended. Nonetheless, it was able to join homogeneous studies together for sensitivity analysis, with a view to meta-analysis.

### Data collection

The following inclusion criteria were assessed: how the randomization was carried out, blinding, whether the intervention was defined and what descriptions were provided for the experimental group and control group.

The information gathered on the included studies consisted of detailed descriptions of the methods, participants, interventions, outcomes, numbers and reasons for withdrawals.

The methodological quality was assessed in order to identify potential sources of bias.

### Statistical analysis

The outcome data were extracted and the odds ratio (OR) and 95% confidence intervals (CI) were analyzed for individual studies, considering only the patients who had had systemic allergic reactions to insect stings or challenge.

To assess the heterogeneity between studies, chi-square tests were performed. In these, a P value < 0.05 indicated significant differences between studies and raised questions regarding whether the results could be meaningfully combined.

### Description of studies

The search for articles in the different databases, using the predetermined key words, resulted in 2,265 articles. The search through bibliographic references added another eight articles. After the first selection, 178 studies remained. Through the second and more rigorous filter, only four controlled studies that were published between 1996 and 2008 were found to satisfy all the inclusion criteria. The methodologies, participants, interventions and results of the studies included are listed in Table 1.^7-10^ Overall, 306 participants were involved in the four selected studies. The participants’ ages ranged from two to 65 years. Only one study compared ant venom immunotherapy with placebo, while three studies compared bee and wasp immunotherapy with placebo or patient follow-up. The main reasons for excluding 174 studies were non-randomization and the lack of proper controls.

**Table 1.** Characteristics of included studies

	Hunt et al.^7^	Schuberth et al.^8^	Valentine et al.^9^	Brown et al.^10^
Participants	History of generalized allergic reactions after insect stings and positive skin test.	History of generalized allergic reactions with cutaneous manifestations after insect stings and positive skin test.	One or more systemic insect sting reactions confined to the skin and positive skin test.	History of grade II-IV hypersensitivity to ant and positive skin test.
Exclusion criteria	Ambiguous histories and negative skin test.	Children with more serious cardiovascular or respiratory symptoms.	Signs or symptoms of more severe reaction (hoarseness, dyspnea, bronchospasm or cardiovascular involvement).	History of hypertension, heart disease, poorly controlled lung disease, ACE inhibitor or beta-blocker therapy.
Diagnostic criteria	Generalized allergic reactions and positive skin test	History of systemic insect sting reaction, positive venom skin (honeybee, yellow jacket, yellow hornet, *Polistes*) and IgE antibodies	History of systemic insect sting reaction, positive venom skin (honeybee, yellow jacket, yellow hornet, white faced hornet and *Polistes*)	History of systemic insect sting reaction (grade II-IV) and a positive skin test.
Age	All ages	3 to 16 years	2 to 16 years	17 to 65 years
Percent males	Groups were matched for age and sex	Treatment: 68.7% No treatment: 71.4%	Treatment: 70% No treatment: 75%	Treatment: 63% Placebo: 62%
Interventions	Specific venom immunotherapy: group I (n = 19) Whole-body insect extract immunotherapy: group II (n = 20) Placebo (histamine solution): group III (n = 20)	Specific venom immunotherapy (n = 32) No treatment (n = 42)	Specific venom immunotherapy (n = 45) No treatment (n = 61)	Specific ant venom immunotherapy (n = 35) Immunotherapy with placebo (n = 32)
Outcomes: % systemic reactions after sting challenge (systemic reactions)	Group I: 5% (1) Group II: 64% (7) Group III: 58% (7)	Treatment group: 5.9% (1) No treatment group: 17% (8)	Treatment group: 4.2% (1) No treatment group: 25.9% (7)	Treatment group: 3% (1) Placebo group: 72% (21)
Odds ratio (CI)	Group I versus III: 0.04 (0.01 < OR < 0.68) Group I versus II: 0.09 (0.01 < OR < 0.62) Group II versus III: 1.09 (0.57 < OR < 2.10)	0.35 (0.05 < OR < 2.56)	0.16 (0.02 < OR < 1.21)	0.04 (0.01 < OR < 0.28)
Notes	Drop out: 0	Drop out: 0	Drop out: 0	Drop out: 0
Allocation concealment	B	B	B	A

### Methodological quality

In accordance with the Jadad scale, the methodological quality of the trials included was as follows: one study received the maximum grade of five, according to the quality scores, and three studies received grade three. The articles excluded could not be classified because the published data did not include any detailed assessments. With regard to blinding of the participants, which is an important factor for reducing bias in controlled and randomized trials, only one study was double-blind and three were single-blind (Table 2).^7-10^

**Table 2.** Methodological quality of the included studies according to Jadad score

Study	Jadad score
Hunt et al.^7^	3
Schuberth et al.^8^	3
Valentine et al.^9^	3
Brown et al.^10^	5

## RESULTS

The article search in the databases, using the predetermined key words, resulted in 2,273 articles. The search through the bibliographic references contributed another eight articles. After an initial analysis of titles, abstracts and complete texts, only 178 articles remained. Among these, after a second, more rigorous selection, only four studies matched all the inclusion criteria for a meta-analysis.

The study by Hunt et al.^7^ was the first trial on immunotherapy using insect venom, and it compared bee venom use with placebo and with whole-body extract in a single-blind manner. At the end of the treatment, the venom reactions were tested by insect challenge and the authors found the following systemic reactions:

– Group I (venom): One patient was unable to achieve tolerance of an adequate venom dose before the study ended and only one patient exhibited urticaria after sting challenge.
– Group II (whole body) and Group III (placebo): There were no adverse reactions to immunotherapy. Ten patients presented systemic reactions of urticaria type, two patients presented generalized itching and skin rash and three patients experienced hypotension after sting challenge.

The odds ratios of each group for systemic reactions after sting challenge were:

– Group I versus Group III: OR = 0.10 (0.01 < OR < 0.68)
– Group I versus Group II: OR = 0.09 (0.01 < OR < 0.62)
– Group II versus Group III: OR = 1.09 (0.57 to 2.10)

These data indicate that, concerning the reaction risk, the patients who were treated with the whole-body extract did not differ from those treated with placebo. However, the results presented by specific immunotherapy with *Hymenoptera* venom, in relation to placebo and whole-body extract, proved that this was a safe and effective therapeutic method for patients who presented with severe allergic reactions after insect stings. It was not possible to determine the relationship between the quantity of IgG antibodies and protection because there was no statistical difference between the groups.

In the study by Schuberth et al.,^8^ only children with non-severe systemic reactions were selected to receive insect venom immunotherapy (bee or wasp) or no treatment. The treated and untreated groups presented approximately the same specific IgG levels for venom, and these values decreased in the untreated group during the study (7.68 ± 15.9 to 2.1 ± 4.14 μg/ml).

The authors tried to identify a correlation between predictive factors such as age, gender, skin test or IgE level with reactions. However, no correlation between these parameters was identified. After the end of the immunotherapy, the authors chose not to perform sting challenges, but to follow up potential accidental stings. The systemic reaction ratios per sting and per stung patient were low in both groups, and the difference was not statistically significant. No reaction more severe than the original one occurred in the treated group, and only one of the eight systemic reactions in the untreated group was as severe as the original one. In a comparison between the groups, we obtained the odds ratio value of 0.35 (0.05 < OR < 2.56), which was non-significant.

In the third selected study, Valentine et al.^9^ performed a trial similar to the previous one, among children who presented reactions restricted to the skin (urticaria and angioedema). The patients were randomized to receive immunotherapy with insect venom (bee or wasp) or not. The analyses on the characteristics of the two groups did not reveal any differences relating to age, gender, IgE or specific IgG levels or even to skin test results. With regard to systemic reactions after the sting challenge, there was only one case of a systemic reaction in the group treated with immunotherapy. In the untreated group, the great majority of the reactions were milder than the reactions presented previously, while two were of similar severity and none of them were more severe. The odds ratio obtained in our analyses was 0.16 (0.02 < OR < 1.21). From this information, we believe that immunotherapy for children with reactions restricted to the skin is unnecessary.

The only double-blind, placebo-controlled study was performed by Brown et al.^10^ In the study, patients of all ages with severe systemic allergic reactions to ants (*Myrmecia pilosula*) were selected. After one week with two successive maintenance doses, insect challenges were performed. The systemic reactions presented in the placebo group were: seven patients with grade I, three with grade II, three with grade III and eight with grade IV. In the specific immunotherapy group, there was just one patient with a grade I systemic reaction. From these results, the odds ratio value was 0.04 (0.01 < OR < 0.28), thus proving that the procedure was an effective treatment.

After performing the chi-square test (chi-square for heterogeneity = 0.24; p = 0.623) with the aim of evaluating the heterogeneity of the studies, only two studies^8,9^ were found to be homogeneous with each other. As mentioned earlier, the study by Schuberth et al.^8^ (1983) showed an OR for systemic reactions after accidental stings of 0.35 (0.05 < OR < 2.56), while the study by Valentine et al.^9^ showed a calculated OR of 0.16 (0.02 < OR < 1.21). After meta-analysis on these two studies, the OR became 0.29 (0.10 < OR < 0.87) (Table 3).

**Table 3.** Meta-analysis on two studies^8,9^ comparing *Hymenoptera* venom immunotherapy (experimental group) versus control group. Presentation of the results from the systemic reactions caused by the provocation test or an accidental sting after ending the immunotherapy, according to odds ratios and 95% confidence intervals

Meta Online					
Groups					
Studies	Experimental		Control		Peto OR
	n	n	n	n	(95% CI; random model)
Schuberth et al.^8^	1	17	8	47	0.35 (0.05 to 2.56)
Valentine et al.^9^	1	24	7	27	0.16 (0.02 to 1.21)
Total	2	41	15	74	**0.29 (0.10 to 0.87)**
	5%		20%		

OR = odds ratio; CI = confidence interval.

## DISCUSSION

This is the second systematic review performed to assess the level of evidence for the use of specific immunotherapy with insect venom among patients with systemic allergic reactions. The previous systematic review^11^ evaluated eight articles, but it also included observational studies. The authors of the previous review concluded that the findings showed that specific immunotherapy was effective for treating hypersensitivity to *Hymenoptera* venom.

Our review therefore differed from the previous one by adopting methodology for systematic reviews that only considered randomized clinical trials to be within the inclusion criteria. Overall, 2,273 articles on this subject were found in the worldwide literature, using the predetermined key words. From these, 2,269 articles were excluded from this review and just four articles matching all the predetermined inclusion criteria were selected. All of these four articles were written in English. It was found that no randomized controlled trials had been published in other languages.

The statistic analysis for heterogeneity, which took into account the participants’ characteristics, type of study and outcome, was significant for only two studies: Schuberth et al.^8^ and Valentine et al.^9^ This resulted in an OR of 0.24 and p = 0.623. Consequently, because p was greater than 0.05, we could conclude that these two studies were not heterogeneous and could be included in the meta-analysis. The two remaining articles, as demonstrated in Table 1, differed from these two articles for two main reasons: the patients recruited were not children and those patients presented severe reactions (grade II to IV) that were not restricted to the skin.

The reason why there are only a few prospective trials that were placebo-controlled and included subjects that presented hypersensitivity to insect venom probably relates to the severity of the allergic reactions. In many cases, these reactions might be fatal. Specific-venom immunotherapy has been considered by most authors to be highly effective, therefore precluding the use of placebo. This is very evident in the study by Hunt et al.,^7^ which demonstrated clearly that immunotherapy with insect venom was safe and an effective therapeutic method among high-risk patients.

### Strength of evidence

The effectiveness of immunotherapy was evaluated by re-exposure to the cited allergen, through an accidental sting or a sting challenge.

**Figure 1:**
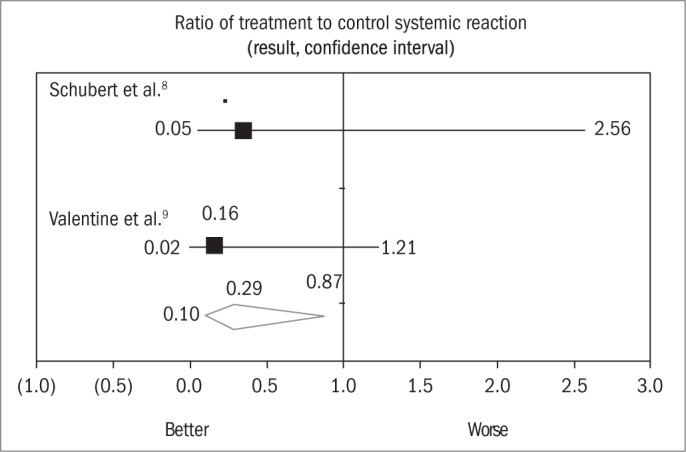
Meta-analysis on two studies^8,9^ comparing *Hymenoptera* venom immunotherapy versus follow-up alone (no treatment), among patients (children) who had only presented skin reactions to the first sting. Presentation of the results from systemic reactions after ending the treatment, according to odds ratio and 95% confidence intervals (Peto's method).

The analysis presented as Figure 1 shows that after performing the meta-analysis on the two studies, the combined odds ratio calculated using Peto's random method for systemic reactions after immunotherapy^8,9^ became 0.29 (CI: 0.10 to 0.87). Taken individually, the odds ratio in the study by Schuberth et al.^8^ was 0.35 (CI: 0.05 to 2.56) and in Valentine et al.,^9^ it was 0.16 (CI: 0.02 to 1.21). Consequently, by performing the meta-analysis on the two studies conjointly, we were able to “increase” the sample size. Thus, the non-significant odds ratios and the respective confidence intervals that included the unit in the separate studies became significant values in combination.

Another possible method for interpreting the data would be to analyze the percentage of systemic reactions (Table 3). After the meta-analysis, we found that in the experimental group (treated), the incidence of systemic reactions was 5%, while in the control group (untreated), the incidence was 20%.

The lack of allocation concealment and the fact that the studies were not double-blind may possibly have resulted in overestimation of the effects of treatment. These aspects of the study design were poorly described. Publication bias is also possible, but few studies were available for determining such occurrences.

### Applicability

The above data indicate that specific immunotherapy is effective among children with systemic reactions restricted to the skin. Nevertheless, when the severity of the reaction that occurred after the sting challenge or accidental sting is taken into account, it is observed that the benefits are non-significant. In the study by Schuberth et al.,^8^ the reactions that occurred after reexposure to stings, not only in the group that received specific immunotherapy but also in the group that did not, were milder than the original ones. These characteristics were also observed in the study by Valentine et al.^9^: 84 stings among the children who received the immunotherapy resulted in only one systemic reaction; while in the group that was not treated, 196 stings resulted in 18 systemic reactions. Even though the number of reactions was significantly higher in the untreated group, 16 of the 18 reactions were milder than the original one and two were similar in severity to the original reaction.

Consequently, since there are no data showing severe systemic reactions involving the respiratory and/or cardiovascular system, there is no need for immunotherapy among children who present only a skin reaction. This was also found in an important study by Golden et al.^12^ that assessed the evolution of hypersensitivity to *Hymenoptera* venom after a time period that ranged from six to 32 years by comparing a group of children that received immunotherapy and another group that did not receive specific immunotherapy. Among the patients who did not receive treatment and who had a history of mild systemic reactions, 87% did not present a systemic reaction after reexposure to the sting. Six patients presented skin reactions, six presented moderate reactions and in no case was the reaction more severe. The authors recommended, backed by their results, that specific immunotherapy should be implemented for children with moderate to severe reactions, but did not indicate such therapy for patients with mild reactions, because the observed risk of severe reactions was lower than 5%.

The other two studies^7,10^ were considered to be trials of good methodological quality, but could not be included in the meta-analysis due to the heterogeneity between them.

The study by Hunt et al. was the first controlled trial in the worldwide literature, and it recommended the use of immunotherapy with purified venom.^7^ The therapy used until that study had been prepared using whole-body extracts from bees and wasps. These authors assessed the allergenic potential of these extracts in laboratories and observed, through histamine release by basophils, that the allergens to which the patients were sting-insect sensitive were not present in the whole-body extract but in the purified venom. The study by Hunt et al.^7^ was undoubtedly a very important milepost within this field, because it compares the use of placebo, whole-body extract and venom for insect venom allergy diagnosis and therapy. The results obtained by these authors demonstrated that treatment with whole-body extract and placebo did not differ between each other, while specific-venom immunotherapy showed effectiveness of 98%.

In the study by Brown et al.,^10^ which was considered to be of high methodological quality because it was double-blind and placebo-controlled, the selected patients presented severe systemic reactions to the venom of the jack jumper ant (*Myrmecia pilosula*), which accounts for approximately 90% of anaphylaxis to insect venom in southeastern Australia. The odds ratio for systemic reactions after sting challenge was calculated to be 0.04 (CI: 0.01 to 0.28), thus demonstrating that specific immunotherapy had a protective effect. Seventy-two percent of the systemic reactions occurred after performing a sting challenge in the placebo group (seven grade I reactions, three grade II, three grade III and eight grade IV), while 3% of the reactions in the immunotherapy group (only one grade I reaction) were systemic. Among the adverse reactions to immunotherapy, 22 of the 64 cases reported by patients were considered to be objective reactions (34%) and, in two cases, there was a decrease in arterial pressure. The substantial risk of a severe reaction to an additional sting among untreated patients was approximately 70% (95% CI: 61-78). This rate was much higher than the 27% to 57% reported for bee or wasp allergy.^13^ One important observation that must be made in this regard is that the study by Brown et al. was the only controlled trial on ant (*Myrmecia pilosula*) venom reactions. It needs to be born in mind that in Brazil^14^ and in the United States^15^, ants are responsible for a large number of accidents, particularly among children, and the most important ant type is *Solenopsis sp*.^14,15^

The hypersensitivity that occurs due to fire ant (*Solenopsis sp*) venom is an increasing and important cause of morbidity and mortality in the United States. In 1989 alone, 32 deaths resulted from accidents with these insects.^16^ Although whole-body extract is ineffective in relation to the venom of bees, wasps and jack jumper ants, this type of extract continues to be used for treating allergies relating to *Solenopsis* *sp* ant venom,^17^ with a very satisfactory clinical response. This is because considerable quantities of venom can be found in the whole-body extract (approximately 20 μg per dose).^18^ There are no double-blind, placebo-controlled studies assessing the effectiveness of these studies. There are only observational studies that have validated immunotherapy with whole-body extract to treat hypersensitivity to the venom from this type of ant.^19-21^

### Other relevant information

Even though the evidence is based only on one trial^7^ and on observational studies,^11,22-25^ the efficacy of specific-venom immunotherapy for avoiding anaphylaxis to bee stings or venom is well accepted. This recommendation can be found in an important manual^26^ published conjointly by three major allergy and immunology societies: the American College and the American Academy of Allergy, Asthma and Immunology (ACAAI and AAAAI); and the Joint Council of Allergy, Asthma and Immunology. This publication recommends immunotherapy for patients who present a history of systemic allergic reaction to *Hymenoptera* venom, as demonstrated by the presence of specific IgE antibodies. For patients who present with an anaphylactic history after an insect sting, especially if there were severe reactions such as airway obstruction or hypotension, immunotherapy must be considered. For children below 16 years of age who present with skin reactions alone, immunotherapy is not normally recommended. For adults who only present reactions restricted to the skin, there is some controversy regarding the treatment, but immunotherapy is usually recommended. The risk of a subsequent systemic reaction decreases to less than 5% and, among individuals who still have reactions, these are of a milder intensity.^27^

In cases in which there is doubt, it is important to assess the risk-benefit relationship of immunotherapy, in an attempt to assess the likelihood of a severe reaction from a future exposure. Consequently, the clinical decision regarding whether to start immunotherapy must include an epidemiological assessment on the natural history.^27,28^ In the case of insect venom allergy, it is firstly important to determine the individual's risk of presenting another allergic reaction when again stung, i.e. the reexposure risk. Secondly, the likelihood of being stung again needs to be assessed: this depends mainly on lifestyle and exposure to the insect. Thirdly, the correct etiological diagnosis needs to be established, with identification of any events that caused sensitization to the venom responsible for the reaction.

The prevalence of systemic reactions after insect stings is low, but the fear of a more severe reaction or even the idea that death might result from the stings may produce a negative effect regarding patients’ emotional state and social activities. In a study by Antonicelli et al.,^29^ the mortality rate due to insect venom allergy was low, but half of those deaths could have been avoided among patients with a history suggestive of allergy, had immunotherapy been performed. Exemplifying this, in an open and controlled study, patients were randomized to receive immunotherapy or were advised to use adrenaline, if necessary (epinephrine autoinjector). A quality-of-life questionnaire was distributed one year before and one year after the immunotherapy or the used of the epinephrine autoinjector. Among the patients who were allergic to wasp venom, there was a clinically significant improvement in those who were treated with immunotherapy, compared with patients who were only advised to use adrenaline if necessary.^30^

## CONCLUSION

Treatment of hypersensitivity to *Hymenoptera* venom with the use of specific-allergen immunotherapy is an important method through which allergists can modify the course of the disease and improve patients’ quality of life, not only from the psychological perspective but also from the objective point of view, by reducing the intensity of systemic reactions.

Specific-venom immunotherapy should be recommended for adults with systemic reactions and for children with moderate to severe reactions, but there is no need to prescribe it for children who only present skin reactions after an insect sting, especially if the exposure is very sporadic. Importantly, the risk-benefit relationship should be assessed in each case.

Little is known about the natural history of hypersensitivity to *Solenopsis sp.* and the effectiveness of immunotherapy, unlike the situation regarding other insects. Whole-body extract of *Solenopsis sp.* has been shown to contain important venom allergens, and evidence supporting the effectiveness of immunotherapy with whole-body extract is continuing to be accumulated over the course of time, in spite of the lack of placebo-controlled trials.
